# Parental Engagement in Infant Obesity Preventative Interventions: Maternal Awareness, Barriers, and Facilitators Identified in a Cross-Sectional Survey

**DOI:** 10.1007/s10995-026-04294-9

**Published:** 2026-08-06

**Authors:** Agnes Gelmini, Cassandra L. Tellegen, Alina Morawska

**Affiliations:** School of Psychology, Parenting and Family Support Centre, The University of Queensland, St Lucia, QLD 4067 Australia

**Keywords:** Infant obesity, Early prevention, Maternal perceptions, Parental engagement, Parenting programs

## Abstract

**Objectives:**

Although close to 1 in 10 children under age 2 gain excess weight and can remain overweight throughout life, engaging parents in early preventative interventions is challenging because they may not recognize the issue of overweight in infancy. This study aimed to inform knowledge about maternal perceptions of infant obesity, to investigate topics of interest for program content and identify barriers and facilitators to parental engagement.

**Methods:**

Mothers (N = 240) of 0 to 24-month-old children completed an online cross-sectional survey.

**Results:**

The majority of mothers were aware that overweight can affect infants, however most considered that excess weight becomes an issue only after 2 years of age and believed that parents should be more worried about their child being underweight than overweight. Mothers were also open to participating in a parenting program, were mostly interested in program content about promoting babies’ health but not feeding, and preferred advertisements for positive parenting and promoting babies’ health rather than preventing infant obesity.

**Conclusions for Practice:**

These findings can help to develop effective strategies to enhance parental engagement and optimize parenting interventions in infant obesity prevention research.

## Significance

Excess weight in infancy is associated with an increased risk of obesity across the life course, yet engaging parents in early obesity prevention remains challenging. Parents may not perceive infant overweight as a concern and may be more focused on preventing underweight. This study adds to the literature by examining mothers’ perceptions of infant obesity alongside their preferences for parenting program content and promotional messaging. Findings suggest that framing interventions around positive parenting and promoting babies’ health, rather than explicitly focusing on obesity prevention, may provide a more acceptable approach to engaging parents in early prevention efforts.

## Introduction

The global rise in childhood overweight and obesity is a pressing public health issue (Lobstein et al., 2015). Children can become overweight as early as six months of age (Harrington et al., 2010), with recent data indicating 9.2% of U.S. infants aged 0–2 years are classified as high weight for length (Ogden et al., 2020). Excess weight during infancy is a strong predictor of obesity later in life (Ward et al., 2017). Contrary to the belief that "baby fat" disappears with age (Eli et al., 2014), early overweight is linked to numerous health issues throughout life (Caprio et al., 2020). Therefore, early prevention, with a focus on parental involvement, is critical given their influence on children’s development and habits (Dietz & Baur, 2022). However, engaging parents in such interventions remains challenging (Byrne et al., 2016).

This paper will also refer to infant overweight using the terms ‘chubby’ and ‘chubbiness’ as research examining obesity-related terminology has found that ‘chubby’ is perceived as one of the most acceptable and desirable descriptors among both parents and children compared with more clinical or potentially stigmatizing terms such as ‘obese’ or ‘fat’ (Gray, et al., 2011; Woo, et al., 2022). A key barrier is the perception of infant chubbiness as a sign of health (Goodell et al., 2008), while underweight raises greater concern (Wright & Weaver, 2007). For example, parents often identify children with higher growth percentiles as the healthiest (Sullivan et al., 2011) and worry less about overweight (Baughcum et al., 2001). However, societal views shift as children grow, with chubbiness later associated with poor health or neglect (Southwell & Fox, 2011). This perception gap underscores why many parents may not recognize early childhood obesity as an issue.

Parental underestimation of their child’s weight is another challenge. Studies show parents often misjudge their child’s weight, especially in younger children (Abbott et al., 2010). This extends to infants, where parents underestimate weight status even when informed by health professionals (Byrne et al., 2016). Moreover, parents often dissociate the risks of excess weight in their own child from those they acknowledge in others (Wen et al., 2010). Without recognizing their child’s risk, parents are unlikely to adopt preventive behaviors (Musaad et al., 2015; Prochaska & DiClemente, 2005).

Beyond perception, strategies to increase parental engagement are crucial, as research and intervention studies often face difficulties in recruiting and retaining participants (Daniels et al., 2012a, 2012b). Parental engagement involves multiple levels, from initial outreach to sustained participation (Morawska & Sanders, 2006). Despite these challenges, parents of infants express a strong interest in improving their skills and knowledge, particularly concerning their child’s health (Campbell et al., 2013). Programs tailored to parents’ needs and motivations, such as addressing feeding practices or readiness to change, have been shown to foster engagement (Love et al., 2018). However, logistical barriers like inconvenient timing or lack of childcare, as well as socioeconomic factors, often hinder participation (Boswell et al., 2019; Mytton et al., 2014).

Despite these findings, there is limited research on effective methods to engage parents in early obesity prevention, particularly regarding communication strategies and content. Effective messaging may play a pivotal role, as parents perceive and respond uniquely to excess infant weight. To address this gap, our study aimed to understand maternal perceptions of infant obesity and explore barriers and facilitators to engaging parents in prevention programs.

We hypothesized that most mothers would be unaware that obesity can affect infants (H1a) and would view excess weight as a sign of health in infants under two years (H1b) but not over two years (H1c). Mothers were also expected to show greater concern for underweight than overweight at both a general (H1d) and personal level (H1e). Regarding program content, we predicted that feeding would be a major topic of interest, particularly portion sizes and nutrition over concerns like overfeeding (H2a, H2b). Additionally, we anticipated that mothers would express higher motivation to participate in general health programs for their infants (H2c) compared to obesity prevention programs (H2d). We planned to control for socioeconomic status (SES) and infant weight status. Finally, we aimed to explore potential barriers to participation in a parenting program focused on infant healthy living.

**Table 1:** Demographic and anthropometric characteristics of 240 mothers with children 0–24 months of age

Characteristics		Subgroup	*n*	*%*
*Infant characteristics*				
*Age*		0 to 6 months	76	31.7
		7 to 12 months	53	22.1
		13 to 18 months	70	29.1
		19 to 24 months	41	17.1
Gender (*n* = 133)^a^		Male	70	52.6
		Female	63	47.4
Weight for-length-percentile category (*n* = 127)^a^		Overweight (≥ 95)	16	12.6
		Normal weight (5 to 94.9)	101	79.5
		Underweight (< 5)	10	7.9
*Maternal characteristics*				
Ethnicity (*n* = 238)^a^		Caucasian Australian	200	84
		Aboriginal/Torres Strait Islander	3	1.3
		South-East Asian	4	1.7
		North-East Asian	4	1.7
		European	17	7.1
		Other	10	4.2
Marital status		Married or de facto	236	98.3
Education level (*n* = 238)^a^		≤ Completed high school	25	10.5
		Trade/technical college	32	13.4
		≥ University degree	181	76.1
Employment status (*n* = 236)^a^		Full time (including maternity leave)	85	36
		Part time	93	39.4
		Home based paid work	10	4.2
		Not working (e.g., home parent, student)	48	20.4
Meeting household expenses (*n* = 238)^a^		Yes	197	82.8
		No	40	16.8
		Not sure	1	0.4
Left-over money (*n* = 237)^a^		Yes, can purchase most of the things they want	103	43.5
		Yes, can purchase only some of the things they want	96	40.5
		Not enough to purchase much of the things they want	38	16
Family structure (*n* = 238)^a^		Original family	231	97.1
		Step family	2	0.8
		Sole parent family	5	2.1
Maternal weight category		Obese (BMI ≥ 30)	37	15.3
		Overweight (BMI 25–29.9)	63	26.3
		Normal weight (BMI 18.5–24.9)	136	56.7
		Underweight (BMI < 18.5)	4	1.7

*BMI*, Body mass index.

^a^*n* differ due to missing data.

## Method

### Measures

Questionnaires were either adapted from existing measures or developed to address gaps in previous literature.

The *Family Background Questionnaire* (FBQ; Sanders & Morawska, 2010) assessed standard sociodemographic characteristics from mothers. Maternal weight status (body mass index, BMI; kg/m^2^) were calculated from self-reported weight and height and were classified as obese (≥ 30), overweight (25–29.9), normal weight (18.5–24.9) and underweight (< 18.5) (World Health Organization, 2000). Infant weight-for-length (WFL) percentiles were based on mothers’ report of current infant weight and length and were calculated using the 2006 WHO Child Growth Standards (< 24 months) (World Health Organisation, 2006). Infant weight status was categorized as overweight (≥ 95th percentile), normal weight (5th-95th percentile), and underweight (5th percentile) (Onis, 2006).

#### Maternal Awareness

We assessed maternal knowledge about infant obesity on a 5-point Likert scale on two statements, c*hildren under 24 months can be overweight* and *children over 24 months can be overweight*. Note that this assessment captured only a specific aspect of maternal knowledge (recognition that overweight can occur in young children) and may not fully reflect the broader, multidimensional construct of maternal awareness regarding infant obesity.

#### Perception of Chubbiness

Maternal perception of chubbiness was investigated with five items on a 5-point Likert scale using four statements (*chubbiness is a good way to tell how healthy a baby is*, *usually thin babies are unhealthy babies*, *usually chubby babies are unhealthy babies* and *chubby babies are healthier than thin babies)* adapted from Boyington and Johnson (2004). A fifth statement assessed maternal knowledge about infant growth (*chubby babies naturally lose their puppy fat as they grow up)*. The words *chubbiness* and *chubby* were used on purpose to match terminology commonly used among parents.

#### Change of Perception About Overweight

We assessed maternal perception of the perceived turning point in children’s development at which excess body fat (chubbiness) may become an issue, based on the question: *at what age do you think that it is not ok for a child to be chubby anymore?* There were 11 response options (*0–4 months, 4–6 months, 6–8 months, 8–12 months, 18–24 months, 2–3 years, 3–5 years, 5–10 years, over 10 years old, never*).

#### Concern About Infant Weight Status

Four items assessed maternal concern about infant weight status. At a general level, mothers were asked to rate two statements: *should parents worry if they have been told by a doctor that their baby (under 24 months) is underweight/overweight?,* using a 5-point Likert-type scale (*not at all, slightly, moderately, very, extremely*). At a personal level, mothers rated how much the following statements: *do you worry that your child might become underweight/overweight in the future?* applied to them. These two latter items were developed by Gross et al., (2011).

#### Topics of Interest for Program Content

We assessed maternal concern and interest related to raising their baby using a 13-item list. Participants were asked to select five major topics of concern or interest. When *feeding* was selected, six further sub-topics were displayed.

**Table 2:** Maternal endorsement of topics of interest for program content (n = 233)^a^

Topics of interest for program content	n	(%)
*Main endorsed topics*		
Promoting baby’s health and wellness	104	44.6
Managing misbehaviour (e.g., throwing tantrums)	100	30.4
Taking care of yourself as a parent	100	42.9
Establishing age-appropriate limits	99	42.5
Sleeping	96	41.2
*Other topics*		
Setting a good example: parents as positive models	89	38.2
Relationship with partner	82	35.2
Dealing with stress or anxiety	74	31.8
Feeding	56	24
Identifying good sources of information	56	24
Baby not being able to keep themselves busy	49	21
Safety at home (e.g., childproofing your house)	42	18
Crying	33	14.2
*Feeding sub-topics (n* = *56)*		
Developing good eating habits	42	75
Appropriate portion sizes, nutritional value of foods	38	67.9
Dealing with food refusal or fussiness	37	66.1
Creating a positive environment at meal times	20	35.7
Recognizing hunger and fullness in your baby	17	30.4
Consequences of overfeeding or poor feeding on baby’s health (e.g., diabetes, obesity)	8	14.3

^a^*n* differ due to missing data.

#### Motivation to Engage

One question assessed maternal motivation to participate in a program (*If a free 2-h discussion group focusing on strategies to promote your baby’s health and wellness was available, would you be interested in attending it?*). Response options were *yes*, *no*, *not sure*.

#### Program Messaging

Five items investigated promoting a parenting program to prevent infant obesity. Mothers who had answered *yes* or *not sure* to the question about attending a program were asked to imagine reading an advertisement for a free parenting intervention in a newspaper. Mothers rated five suggested titles in terms of how interested they would be in each program using a 5-point Likert scale (*not at all* to *extremely*).

**Table 3:** Mothers’ reported interest in program advertisements (n = 196)^a^

Program titles	Not at all interested (%)	Slightly (%)		Moderately (%)	Very (%)	Extremely interested (%)
Positive parenting (*n* = 195)^a^	4.1	17.4	20		35.4	23.4
Hassle free mealtimes	20.9	26	27		15.3	10.7
Baby’s health and wellness	2.6	21.4	29.6		30.1	16.3
Positive feeding practices	9.7	16.8	30.6		28.1	14.8
Preventing infant obesity	20.9	25	18.9		23	12.

^a^*n* differ due to missing data.

#### Barriers to Participation

Mothers rated a list of 10 potential barriers how much each barrier would prevent them from attending the program *not a problem at all* to *extremely problematic*.

**Table 4:** Mothers’ reported perception of barriers to attendance to a program (N = 240)

Barriers	Not at all/Slightly problematic (%)^a^	Moderately (%)	Very/Extremely problematic (%)^a^
Financial cost (*n* = 238)^b^	52.1	20.6	27.3
No access to childcare (*n* = 238)^b^	40.3	20.2	39.5
Lack of time	25.4	27.5	47.1
Inconvenient location (*n* = 239)^b^	24.7	22.6	52.7
Conflict with other activities	41.3	30.4	28.3
Inappropriate content of the program (*n* = 238)^b^	47.9	21.4	30.7
Feel uncomfortable accessing a parenting program	89.5	8	2.5
Partner or extended family not supportive (*n* = 238)^b^	94.1	2.9	5.9
Transport difficulties (*n* = 238)^b^	84	5.5	10.5
Do not feel the need to attend a program (*n* = 237)^b^	62	18.6	19.4

^a^Categories have been collapsed.

^b^*n* differ due to missing data.

#### Procedure

Ethical approval was granted in accordance with the guidelines of the University of Queensland and the Australian National Statement on Ethical Conduct in Human Research. Participation in this study was voluntary and anonymous. The study was advertised through childcare centres, playgroups and family associations across Australia.

#### Statistical Analyses

Data were analyzed using IBM SPSS 20. Responses for maternal awareness and perception of chubbiness were collapsed into "disagree" and "agree," excluding the mid-point. Paired-samples t-tests compared maternal perceptions of infant overweight before and after 2 years and concerns about infant weight status at general and personal levels. Descriptive statistics assessed program content interests, with Chi-square tests controlling for infant age as a confounder. Frequency distributions and t-tests explored maternal motivation, preferences, and participation barriers.

Of 340 respondents, 87 were excluded for not specifying their relationship to the child, three for not being mothers, and nine for reporting a child’s age unknown (n = 5) or over 24 months (n = 4). The final sample included 241 participants. Data were screened for missing values and outliers; one case with over 20% missing data was excluded. The mean percentage of missing values was 2.55% (SD = 3.52), with all variables having none or up to two missing values. Analyses employed SPSS pairwise exclusion of missing data.

## Results

### Participants

Participants in this cross-sectional study were 240 mothers with children aged 0 to 24 months old who completed an online survey. Demographic and anthropometric characteristics are provided in Table 1. Eighty-four percent of participants were Caucasian Australian, 76.1% possessed a university degree and 82.8% reported no financial difficulties. Maternal age ranged from 20 to 47 (*M* = 32.97; *SD* = 6.58). All participants were the biological mother of the child except for one foster mother. Mothers who were overweight or obese constituted 41.6% of the sample.

Mean infant age was 11.3 months (*SD* = 6.55) with about half of the children being less than 1 year of age (53.8%). Due to an error in data collection, child gender was only recorded for 127 cases, of which 70 were male and 63 female. In this sub-sample, nearly 13% of children were overweight and 8% underweight.

#### Maternal Awareness

Contrary to our first hypothesis (H1a), mothers in general (70.8%) agreed that infants under 24 months can be overweight, while 21.6% disagreed with the statement. A paired-samples *t*-test showed that mothers agreed more strongly with the statement that children older than 2 years can be overweight (*M* = 4.64, *SD* = 0.76) than with the statement that children under 2 years can be overweight (*M* = 3.76, *SD* = 1.35; *t* (239) = −10.90, *p* <.001). Cohen’s *d* = 0.71, indicated a medium to large effect size. Similarly, the proportion of mothers who disagreed dropped from 21.6% for the first statement (*children under 24 months can be overweight*) to 3.7% for the second statement (*children over 24 months can be overweight*).

#### Perception of Chubbiness

Hypothesis H1b was partially supported. Less than a third of mothers (30.9%) agreed that chubbiness is a good way to tell that a baby is healthy. A large majority of participants disagreed that *chubby babies are unhealthy babies* (77.9%) and also disagreed that *thin babies are unhealthy babies* (65.8%). When agreeing with the later items, mothers were nearly three times more likely to agree that thin babies are unhealthy babies (15.8%) compared to chubby babies (5.4%). Furthermore, 65% of mothers agreed that *chubby babies naturally lose their puppy fat as they grow up*.

#### Change of Perception About Overweight

As predicted (H1c), most mothers (74.1%) considered that chubbiness becomes an issue after 2 years of age and above, while 17.9% of mothers thought that excess weight is already an issue in infancy, of which only 2.9% indicated that it was an issue in the first year of life. The modal value for the age at which mothers deemed chubbiness to be an issue was 3–5 years old with nearly a third of mothers (31%) selecting these ages. The majority of the sample (69%) had situated the turning point at which chubby is not acceptable anymore in a child’s development by the age of 5 and a minority (5.9%) considered that it is never an issue for a child to be chubby.

#### Concern About Infant Weight Status

We hypothesized that participants would consider that parents in general should be more worried about their baby being underweight than overweight (H1d). A paired-samples *t*-test confirmed that although the difference was small (Cohen’s *d* = 0.13), parental concern about infants being underweight received higher scores (*M* = 3.04, *SD* = 0.92) than concern about overweight (*M* = 2.93, *SD* = 0.94; *t* (239) = 2.03, *p* =.043).

When investigating maternal views at a personal level, paired samples *t*-tests revealed that our hypothesis (H1e) was rejected since there was no significant difference in scores for the item *do you worry that your baby might become underweight in the future?* (*M* = 1.55, *SD* = 0.88) and *do you worry that your baby might become overweight in the future?* (*M* = 1.67, *SD* = 0.86; *t* (238) = −1.62, *p* =.106). Mothers in our sample reported being equally unconcerned about their child becoming underweight as overweight.

Post-hoc analyses were performed to compare mothers’ views at a general level and at a personal level. A series of paired-samples *t*-tests revealed that mothers judged that parents in general should be nearly twice as worried about their child being underweight than they were worried about their own child becoming underweight (*M* = 3.03, *SD* = 0.92; *M* = 1.55, *SD* = 0.88, respectively; *t* (238) = 19.06, *p* <.001). Cohen’s *d* showed a large effect size (1.23). Similarly, participants reported being significantly less worried about their own child becoming overweight (*M* = 1.67; *SD* = 0.86) than how concerned they thought parents in general should feel about their child being overweight (*M* = 2.92; SD = 0.94; *t* (238) = 16.30, *p* <.005) (*d* = 1.05).

#### Topics of Interest for Program Content

Table 2 shows the topics of concern and interest endorsed by mothers about raising a baby. Contrary to our hypothesis (H2a), infant *feeding* was not among the five main topics of concern. The most rated topic was *promoting baby’s health and wellness*, followed by *managing misbehaviour*, *taking care of themselves as a parent*, *establishing age-appropriate limits* and *sleeping*. *Feeding* was chosen by less than a quarter of mothers.

In support of our next hypothesis (H2b), of the 56 participants who selected feeding, more than two thirds subsequently selected *portion sizes and nutritional values of food*. A large majority of mothers was also interested in *developing good eating habits* and in *dealing with food refusal or fussiness*. The least popular feeding topic was *consequences of overfeeding or poor feeding on baby’s health*.

A series of Chi-square tests for independence revealed significant relationships between infant age and the following topics of concern: *crying*, *χ*^*2*^(1, *n* = 233) = 12.88, *p* <.001, *phi* = 0.25, *sleeping*, *χ*^*2*^(1, *n* = 233) = 18.69, *p* <.001, *phi* = 0.29, *establishing age-appropriate limits*,* χ*^*2*^(1, *n* = 233) = 21.60, *p* <.001, *phi* = −0.31, and *managing misbehaviour*, *χ*^*2*^(1, *n* = 233) = 18.10, *p* <.001, *phi* = −0.29. Mothers of infants aged 0–12 months were more likely to select *crying* and *sleeping* (22% and 54.3%, respectively) than mothers of older children (4.7% and 25.5%, respectively), while mothers of infants aged 13–24 months were more interested in *establishing age-appropriate limits* and *managing misbehaviour* (59.4% and 58.5%, respectively) than mothers of younger children (28.3% and 29.9%, respectively).

#### Motivation to Engage

Our hypothesis (H2c) was supported, since a little less than half of the participants (42.9%) were interested in a free 2-h parenting program to promote their baby’s health and wellness, 39.2% were unsure and 17.9% were not interested in attending such a program. Overall, 82.1% of mothers were either motivated or undecided about attending a free intervention.

#### Program Messaging

Consistent with our hypothesis (H2d), a paired-samples *t*-test showed that mothers were significantly more interested in a program advertised as aiming to promote their baby’s health and wellness (*M* = 3.36; *SD* = 1.07) than in a program designed to prevent childhood obesity (*M* = 2.81; *SD* = 1.33; *t* (195) = 5.65, *p* <.001). Cohen’s *d* revealed a small to medium effect size (*d* = 0.41).

Table 3 displays frequencies of interest for each program messaging. Among the three favourite advertisements, the positive parenting intervention was elected by a majority of mothers, followed by programs promoting babies’ health and wellness and positive feeding practices. More than a third of participants were also very to extremely interested in a program to help parents prevent childhood obesity. The infant obesity prevention program and the hassle-free mealtime program were nevertheless the least attractive advertisements with forty-one mothers not at all attracted by either program. By comparison, only five mothers were not interested in the health and wellness program title, eight in the positive parenting program title and 19 in the positive feeding practices title.

#### Barriers to Participation

More than half of the sample reported that the most problematic barriers to attending a program would be an inconvenient location, followed by lack of time and no access to childcare (see Table 4). Most mothers felt comfortable accessing a parenting program.

## Discussion

This cross-sectional study aimed to inform strategies for enhancing parental engagement and optimizing interventions for preventing infant obesity. The first goal was to assess maternal knowledge and perceptions of infant obesity. Results partially supported our hypotheses. Specifically, results did not provide support for mothers’ lack of awareness (H1a), confirmed the timing of the change of perception for overweight (H1c) and partially supported perceptions of chubbiness (H1b) and concern about infant weight status (H1d and H1e).

Although participants were highly educated, many believed infants naturally outgrow baby fat and associated thinness in babies with poor health. Such beliefs suggest that while mothers intellectually recognize infant obesity as an issue, this knowledge does not align with their perceptions of health risks in infancy. This highlights a potential gap between understanding and practical awareness, as many overweight infants remain unrecognized until later in childhood, when the condition is more difficult to address (Harrington et al., 2010).

Mothers in the study were less concerned about their own child’s weight status compared to their perceptions of what other parents should worry about. This aligns with the concept of optimistic bias (Weinstein, 1989), where individuals believe they are less vulnerable to risks. Parents may also use denial to protect their self-esteem and sense of competence (Southwell & Fox, 2011). These factors likely contribute to underestimations of infant obesity risk, making early intervention efforts challenging.

The second aim was to identify barriers and facilitators to parental engagement in obesity prevention programs. Consistent with prior research (Campbell et al., 2013; Goodell et al., 2008), as hypothesised mothers showed interest in programs promoting their baby’s health but were less drawn to those specifically advertising obesity prevention. Contrary to predictions, feeding topics were not a major area of interest, but nearly half of the mothers responded positively to advertisements for healthy feeding practices. This suggests that while mothers may not prioritize weight or feeding concerns directly, they are still receptive to programs that emphasize positive parenting and health promotion.

Messages focusing on infant growth and body size may deter participation if perceived as critical or stigmatizing (Southwell & Fox, 2011). Since overweight children face high levels of stigma, negative messaging could alienate parents (Lawrence, 2010). Effective strategies should emphasize positive parenting and healthy habits rather than focusing on weight or using moralizing tones. Major barriers to engagement included logistical challenges such as inconvenient locations, time constraints, and lack of childcare (Mytton et al., 2014). Addressing these barriers is essential for improving participation rates. Further work collecting qualitative data may better capture contextual factors, barriers, and facilitators influencing implementation.

The study had limitations. The sample was primarily composed of mothers with higher education and stable finances, limiting generalizability. Socioeconomic factors like education and income significantly influence perceptions of and behaviors related to infant overweight (Baughcum et al., 2000; Boyington & Johnson, 2004). Mothers were also recruited through childcare centres and playgroups which may have further limited the generalizability to mothers who may be more engaged in parenting issues. Additionally, the survey’s use of colloquial terms like "chubby’’ may have influenced responses. Furthermore, an error in data collection reduced the ability to account for infant weight-for-length as a confounding variable or to examine associations between infant weight and maternal perceptions of weight. Maternal weight status was also not included as a covariate in the analyses; however, prior research suggests it may influence parental perceptions and engagement. Future studies should examine this association using analytical designs specifically powered for subgroup comparisons.

Despite these limitations, the findings offer important insights. While mothers demonstrated awareness of infant obesity, this knowledge was often incomplete or misaligned with actual health risks. Furthermore, mothers expressed interest in programs promoting positive parenting and health practices, suggesting opportunities for intervention. Rather than suggesting the development of entirely new interventions, these findings may support the adaptation of existing evidence-based parenting and health promotion programs to incorporate obesity prevention content in ways that align with parental preferences and engagement factors. For example, established parenting interventions such as Triple P-Positive Parenting Program (Sanders et al., 2014) may be well-placed to adapt a Discussion Group or Seminar program specifically to address these health practices in parents of infants.

To enhance recruitment and retention, public health messaging should focus on positive, evidence-based themes such as parenting strategies and child wellness rather than directly addressing weight concerns. Communication should avoid stigmatizing language and be sensitive to parental concerns. Programs should also address parents’ main interests while presenting accurate information about infant obesity in a way that feels supportive rather than critical. These findings underscore the need for tailored approaches that combine effective communication, supportive messaging, and logistical support to improve parental engagement in infant obesity prevention efforts.
